# Tunneling between parallel one-dimensional Wigner crystals

**DOI:** 10.1038/s41598-022-08367-x

**Published:** 2022-03-16

**Authors:** R. Méndez-Camacho, E. Cruz-Hernández

**Affiliations:** 1 Facultad de Ciencias, Universidad Autónoma de San Luis Potosí, Av. Chapultepec 1570, Privadas del Pedregal, 78295 San Luis Potosí, México; 2 Coordinación para la Innovación y Aplicación de la Ciencia y la Tecnología, Universidad Autónoma de San Luis Potosí, Sierra Leona 550, 78210 San Luis Potosí, México

**Keywords:** Materials science, Nanoscience and technology, Physics

## Abstract

Vertically aligned arrays are a frequent outcome in the nanowires synthesis by self-assembly techniques or in its subsequent processing. When these nanowires are close enough, quantum electron tunneling is expected between them. Then, because extended or localized electronic states can be established in the wires by tuning its electron density, the tunneling configuration between adjacent wires could be conveniently adjusted by an external gate. In this contribution, by considering the collective nature of electrons using a Yukawa-like effective potential, we explore the electron interaction between closely spaced, parallel nanowires while varying the electron density and geometrical parameters. We find that, at a low-density Wigner crystal regime, the tunneling can take place between adjacent localized states along and transversal to the wires axis, which in turn allows to create two- and three-dimensional electronic distributions with valuable potential applications.

## Introduction

Semiconductor nanowires (NWs) are exciting components to study unique one-dimensional (1D) physics such as the Coulombic strength-dependent electronic charge fractionalization^1^, quantized conductance^2,3^; or the formation of a periodic charge distribution along the wires, known as a Wigner crystal, first predicted by Wigner in 1934^4^ and recently observed^5–7^. In addition to be remarkable systems for basic research, NWs are also key building blocks in the fabrication of nanoscale electronic and optoelectronic devices^8–11^. Some NW-based devices that has been widely explored includes field-effect transistors (FETs)^12–15^, diodes^16^, nano-logic gates^17^, and nanoprocessors^18^.

When NWs are synthesized by self-assembly, usually pillar or in-plane arrays of parallel NWs (PNWs) are produced. In such arrays, or in the integration into the existing planar electronic platforms^11^, the NWs separation can be short enough to permit electron tunneling between them. Furthermore, with the continuous size reduction in the design of smaller and more powerful devices; for example in advanced sub-5 nm nodes in NW-based vertically or laterally stacked gate-all-around FETs^19–21^, the consequences of the closeness of adjacent NWs must be thoroughly analyzed to both prevent undesirable effects and to look for new architecture strategies^22^.

On the theoretical side, even when there exist a number of advanced approaches to investigate the electron-electron ($\text {e-e}$) interaction in nanostructures, usually such formalisms are limited to one or few charge carriers and to the nanometric scale^23–27^. On the other hand, the many-electron interaction in 1D Wigner molecules, and the close related Friedel oscillations, has been studied by using Luttinger liquid theory as well as quantum Monte Carlo and ab initio methods^28–35^. Far less works dealing with the electron interaction between coupled NWs has been published, even when it can be a simple and powerful approach to explore more complex problems such as the strongly interacting topological states in two and higher dimensions^36^. Still, modeling of micron-long semiconductor NWs and the $\text {e-e}$ interaction for electronic densities (*n*) obtained from usual doping levels (10$^{16}$–10$^{19}$ electrons/cm$^{3}$) is a very cumbersome task, impracticable to carry out in a direct way. For such reason, despite the importance of this subject, the interaction of adjacent PNWs with realistic characteristics remains largely unexplored.

Given the difficulty of directly including the many-body forces to calculate the many-body quantum states, we instead use a coarse-grained model that considers two-electron wave-functions while the presence of the other electrons is assumed to only affect the interaction between the two electrons by a Yukawa-like screening. Although simple, the use of a Yukawa-like potential to address the many-body problem has been extensively used in other branches of physics, such as soft matter and nuclear physics and, applied to electron distributions in nanostructures, this approach is able to correctly describe experimental results related to the Wigner molecule onset in semiconductor NWs^37,38^.

In this contribution, we theoretically investigate the electron tunneling between closely spaced micron-long semiconductor PNWs. The resultant electronic distributions are analyzed as a function of the NWs separation (from 1.2 to 10 nm), for some representative NW cross sections (from 15 to 50 nm), as well as three *n*-doping levels in the range from 10$^{17}$ to 10$^{20}$ electrons/cm$^{3}$. Following previous works^37,38^, we consider a Yukawa-like $\text {e-e}$ interaction to deduce a real-space effective potential, which allows us to calculate the NWs electronic distribution. We focus on the cases where a discontinuous charge distribution along the NWs is produced as well as in the selective tunneling between these charged regions.

The rest of the paper is organized as follows. In “Results and discussion”, we present the ground and first excited electronic distributions for $1 \times 2$ and $1 \times 6$ two-dimensional (2D) arrays as well as for a $6 \times 6$ three-dimensional (3D) array of 1-micron-long GaAs/AlGaAs PNWs and its dependence on the main geometric parameters. We also discuss some implications and possible applications of the emerging electronic patterns in the PNWs arrays. In “Theoretical model”, a detailed derivation and some remarks of the model used to generate the electronic distributions are given. Finally, the conclusions are presented in next section.

## Results and discussion

### The effective potential

**Figure 1:**
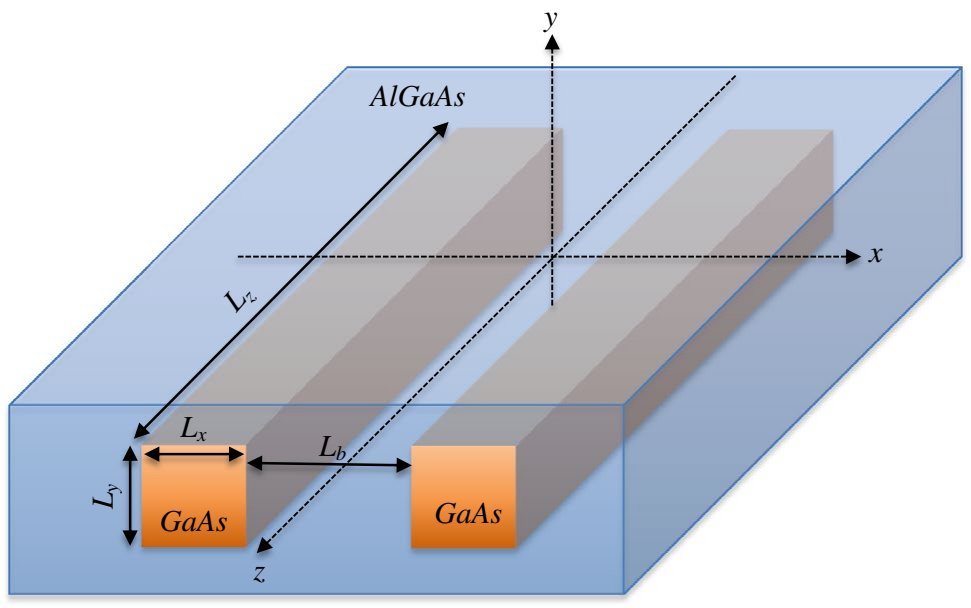
Schematic representation of a $1\times 2$ parallel quantum wires array as the studied in this work: square GaAs wires, with the axis directed along the *z*-direction, are embedded into an AlGaAs matrix and separated by a distance $L_{b}$.

Figure 1 provides a schematic view of one of the systems studied in this work. Such system consists of GaAs PNWs of cross-sectional area $L_{x} \times L_{y}$ and length $L_{z}$, which are embedded in an AlGaAs matrix that acts as a finite potential barrier of width $L_{b}$. As described in detail in “Theoretical model”, we solve the time-independent Schrödinger equation for a spinless two-electron wave function, $\Psi _{1,2} (r)$, considering a Yukawa-like $\text {e-e}$ interaction. Then, by using Fourier transforms, we derive for the *z* component a real-space effective potential able to manage long $L_{z}$ and high *n* values in an easy way. For the *x* and *y* components we consider the usual solutions for *m*-coupled finite quantum wells. The electronic density distributions shown hereafter are then obtained from the square of the calculated $\Psi _{1,2} (r)$.

### Two parallel nanowires

**Figure 2:**
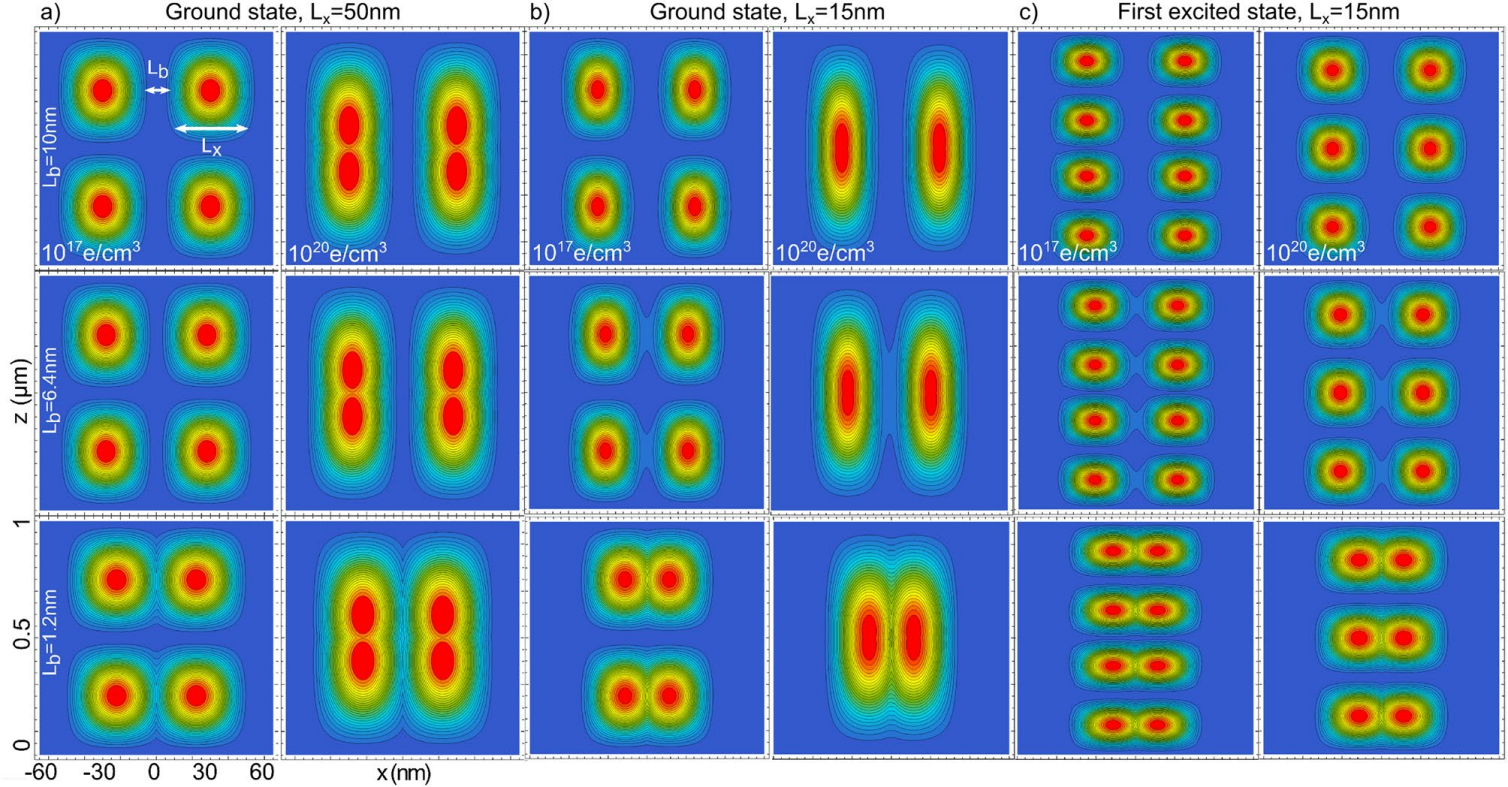
z–x projections of the charge distribution probability for two $1\,\upmu$m-long GaAs/AlGaAs PNWs. The plots correspond to: (**a**) ground state for $L_{x,y}=50$ nm, (**b**) ground state for $L_{x,y}=15$ nm, and (**c**) first excited state for $L_{x,y}=15$ nm. Two different *n*-doping concentration are plotted: $n= 10^{17}$ electrons/cm$^{3}$ and $n= 10^{20}$ electrons/cm$^{3}$. Each row corresponds to a different $L_b$.

In Fig. 2a, we plot the z–x proyection of the ground state distributions for two PNWs of size $L_{x,y}\equiv L_{x}=L_{y}=$ 50 nm and $L_{z}=1 \,\upmu$m, kept apart by a variable $L_b$, for $n= 10^{17}$ electrons/cm$^{3}$ (left) and $n= 10^{20}$ electrons/cm$^{3}$ (right). The ground and first excited state for a similar system, with a smaller cross-section $L_{x,y}=$15 nm, are presented in Fig. 2b,c, respectively. Each row in Fig. 2 corresponds to a different $L_b$ separation. We can observe from the upper row in Fig. 2 (where there is not tunneling between NWs), that in agreement with previous reports on individual NWs^37,38^, the lower *n* concentration produces for the ground state well defined individual distributions along the NWs while for the higher concentration the electrons merge in a single distribution. This concordance is also observed in the first excited state.

For the ground state, we can observe from Fig. 2 that tunneling is strongly dependent on the electron density. Such dependence is mainly due to the localized and extended states induced for the relative low and high densities, respectively. For the excited states, a localized distribution is always found. Given the energy difference between the confined levels (meV for *x*-*y*, and $\upmu eV$ for *z*), the excited states plotted in Fig. 2 are those related to the *z*-component, while the *x* and *y* components remains in the ground state.

**Figure 3:**
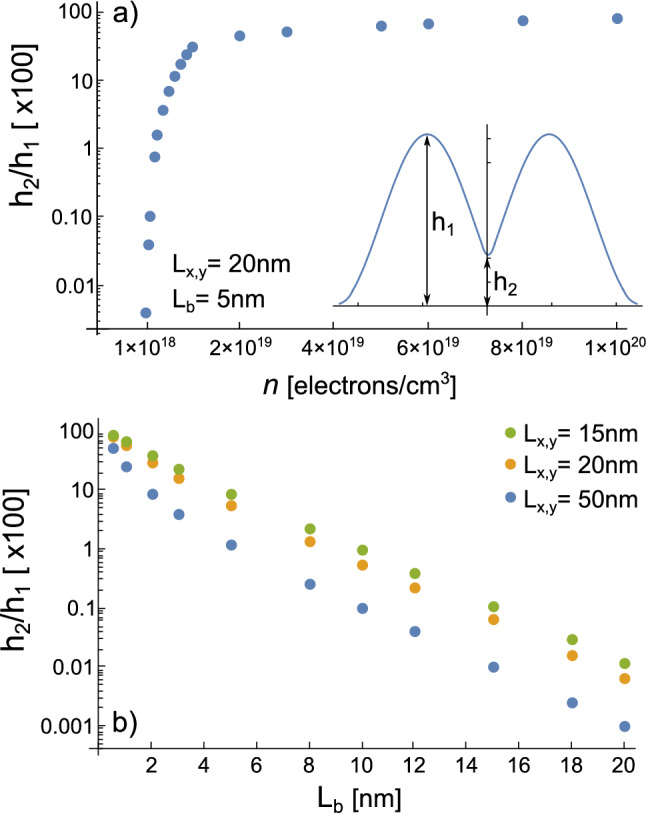
(**a**) $h_2/h_1$ dependence on the electron density *n* for an array of $1 \times 2$ PNW with $L_{x,y}=20$ nm and $L_b=5$ nm. (**b**) $h_2/h_1$ dependence on $L_b$ for a similar array for three different cross sections $L_{x,y}$. We use the $h_2/h_1$ ratio as a parameter to describe the tunneling strength between localized distributions. Here $h_1$ and $h_2$ are heights taken from 2D electron distribution profiles as illustrated in the inset in (**a**). In (**a**), the profiles are taken along the *z*-axis from one of the NWs while in (**b**), the profiles are taken across the NWs along the *x*-axis.

As shown in Fig. 2, the variation on the NWs cross-section affects the way the tunneling take place. For $L_{x,y}=$15 nm, electrons are less contained by the AlGaAs barriers, so the tunneling takes place for a larger $L_b$ separation as compared to the PNWs of cross-section $L_{x,y}=$50 nm. According to our model, $L_b$ and $L_{x,y}$ are the main parameters to control the tunneling strength through the PNWs, while by modifying *n* one can control the electron distribution connection along each wire. The dependence of the tunneling strength on these parameters, for the ground state in an array of two PNWs, are presented in Fig. 3a,b in terms of the $h_2/h_1$ ratio where $h_1$ and $h_2$ are heights in the 2D density profiles taken as shown in the inset of Fig. 3a. From these figures, we can observe that an appreciable tunneling where $h_2 \sim 0.1h_1$ for such configuration is given for values of $n \sim 6 \times 10^{18}$ electrons/cm$^{3}$ (along the wires) and $L_b \sim 5$ nm for $L_{x,y}$ = 15 nm or $L_b \sim 2$ nm for $L_{x,y}$ = 50 nm (transversal to the wires). As we show further below, for 2D and 3D arrays of more than two PNWs, the tunneling strength is not uniform between the NWs, so there is not simple relationships for such cases as the shown in Fig. 3.

### Multiple parallel nanowires

**Figure 4:**
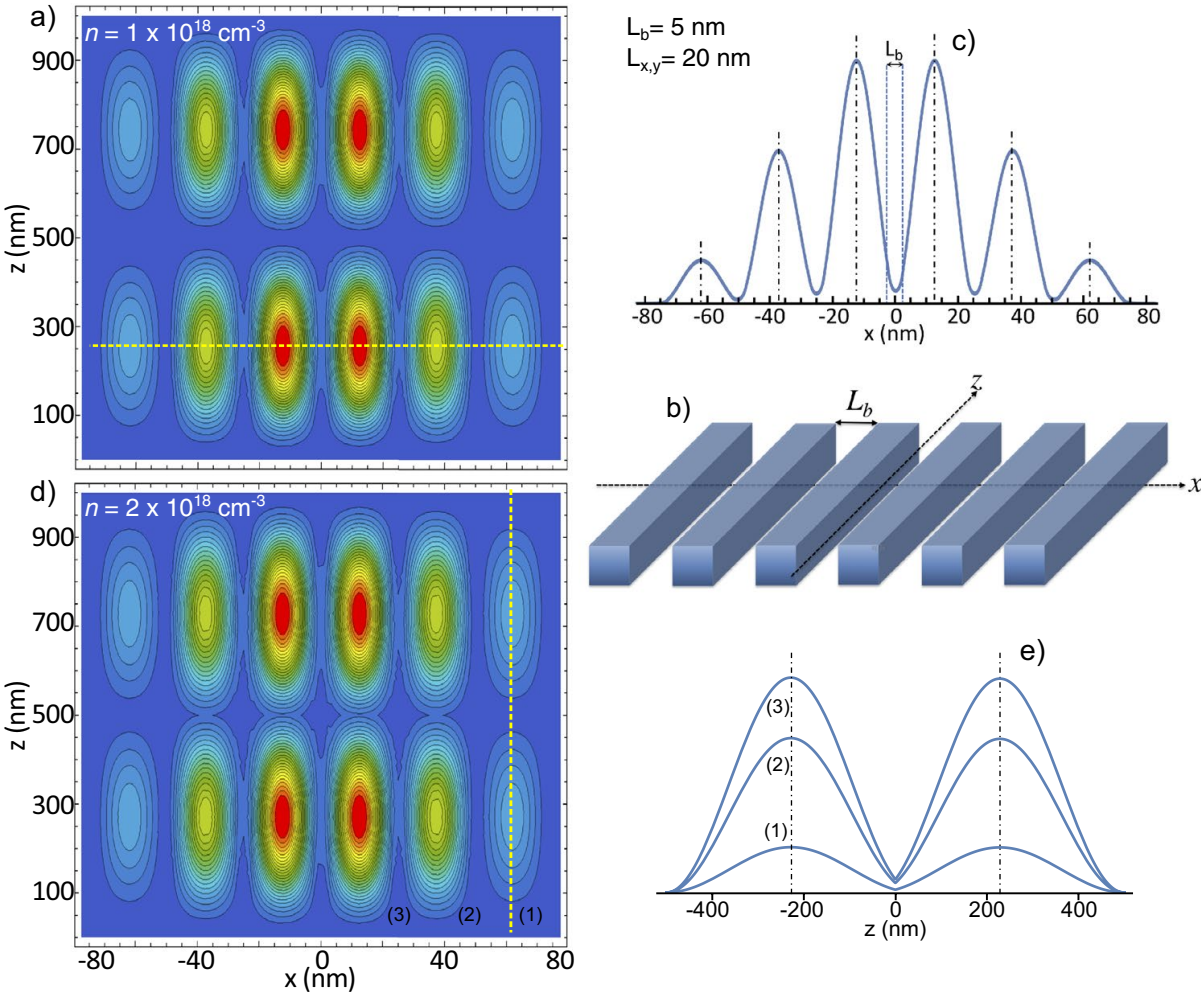
(**a**) Ground state charge distribution for an array of six 1-micron long PNWs of cross section $L_{x,y}=20$ nm, $n= 1 \times 10^{18}$ electrons/cm$^{3}$, and mutual separation $L_{b}=5$ nm. (**b**) Illustrative representation of the $1\times 6$ GaAs/AlGaAs PNWs array. (**c**) Profile of the electronic density taken along the horizontal yellow line indicated in (**a**). In (**d**), *n* is increased to $2 \times 10^{18}$ electrons/cm$^{3}$ in order to trigger the connection between the charge distributions along the *z*-axis. In (**e**), three profiles taken along the *z* direction from (**d**) are plotted; the vertical yellow line in (**d**) indicates where the profile number (1) was taken.

Additional interesting properties comes out in systems composed of more than two PNWs. As an example, the electronic ground state distribution in a $1\times 6$ PNWs array, as the one depicted in Fig. 4b, is plotted in Fig. 4a. The relative low *n* concentration ($1 \times 10^{18}$ electrons/cm$^{3}$) is used to induce the formation of localized electronic states along the NWs *z*-axis (a Wigner molecule). We set a small cross-section ($L_{x,y}=20$ nm) and a short distance between NWs ($L_b=5$ nm) to observe a strong tunneling between them. Profiles taken along the *x*-axis presents a notorious intensity modulation from wire to wire, as the shown in Fig. 4c (taken along the horizontal yellow line drawn in Fig. 4a). Even when this modulation is not observable in the two PNWs system (see Fig. 3), such kind of distributions where the intensity is maximal at the center of a finite number of quantum wells is usually found in common 1D superlattices (see for example reference^39^).

If, in the parameters used in Fig. 4a, *n* is increased to $2 \times 10^{18}$ electrons/cm$^{3}$, a connection between the individual electronic distributions along the NWRs axis can be also established (see Fig. 4d). As the lateral tunneling depopulate the more external NWs, the distribution along the *z* axis is different from wire to wire. This is clear from Fig. 4e, where profiles taken along the *z*-axis from three consecutive NWs in Fig. 4d are displayed.

**Figure 5:**
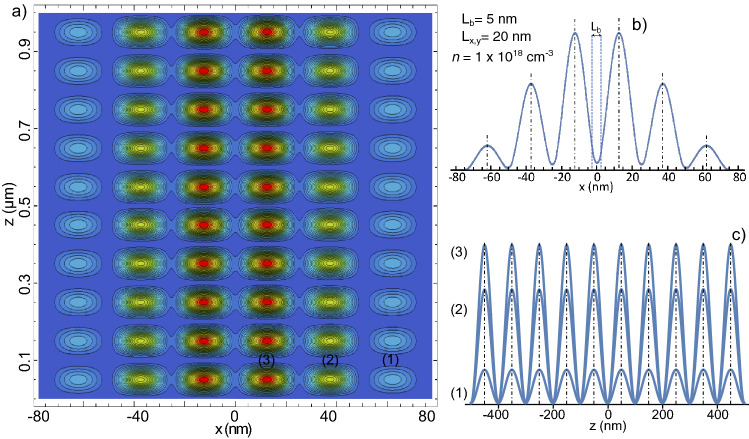
(**a**) Fourth excited state charge distribution for an array of six 1-micron long PNWs of cross section $L_{x,y}=20$ nm, $n= 1 \times 10^{18}$ electrons/cm$^{3}$, and mutual separation $L_{b}=5$ nm. (**b**) Profile of the electronic density taken along the *x* direction. (**c**) Profiles taken along the *z* direction at the *x* positions where the electron density is maximal; each number indicates the place where the profiles were taken from (**a**).

Usually, quantum tunneling is considered only as a 1D problem. The 2D and 3D tunneling cases are addressed as 1D independent tunneling along each spatial direction, without accounting for alterations in the 2D or 3D electron distributions. However, as described before, we found that the electronic tunneling along the transversal direction of the PNWs is actually able to significantly change the electronic distribution along the NWs axis. These effects are more noticeable for higher excited states, where a greater number of localized distributions along the wires emerges.

Because confinement along the *z* axis is very weak and the energy separation between quantized energy levels is very small, of the order of $\upmu eV$, these states can be easily populated and then are particularly important to analyze. As an example, in Fig. 5a we plot the fourth excited state corresponding to a system with the same parameters that the plotted in Fig. 4a. A typical profile, taken along the *x* direction where the density is maximal, is shown in Fig. 5b. By the nature of our model, this transversal profile is practically the same as the shown in Fig. 4c, with the only difference that its absolute intensity is smaller (as the electron density is distributed in more zones along the wires). In Fig. 5c, the profiles taken along the *z* axis show a sharp distribution of the well separated electronic regions.

### 2D and 3D interconnected distributions

As discussed before, three main electronic distributions appears in the arrays of PNWs by modifying *n*, $L_b$, and $L_{x,y}$. When $L_b$ is large enough to block tunneling and *n* is low enough to trigger the Wigner crystallization, then a disconnected but ordered 2D charge distribution is established. However, if $L_b$ is small enough to allow lateral tunneling, then a 2D array of superlattices interconnected along the *x* axis (as in Figs. 4a, 5a) is produced. Furthermore, if the *n* density value allows to connect the charge distributions along the *z* axis (as in Fig. 4d), then a fully connected 2D arrangement can be assembled. Because the NWs *y*-component is mathematically equivalent to the *x*-component considered in this work, the 3D distributions are a straightforward generalization of the 2D distributions previously discussed.

**Figure 6:**
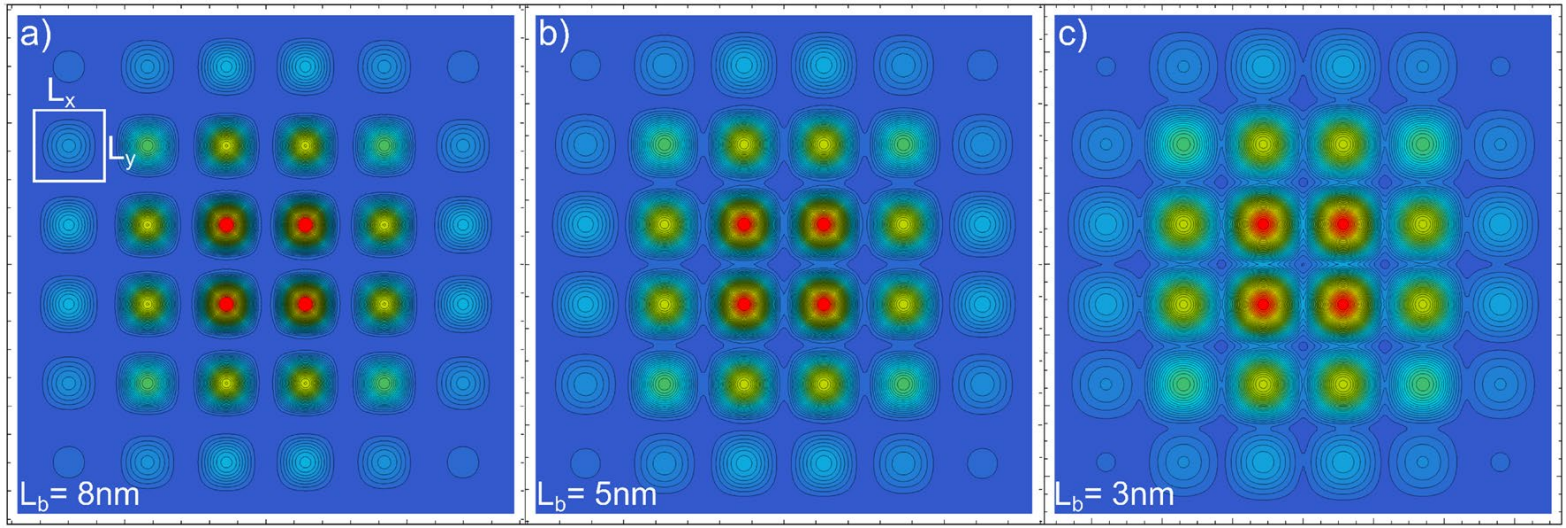
*x*–*y* projections of the ground state electron configuration in $6 \times 6$ PNW arrays of $L_{x,y}=$20 nm. The NWs mutual separation $L_b$ is modified from 8 to 3 nm to observe the gradual transition to a strongly interconnected 2D distribution. The distributions are obtained by considering only the *x* and *y* wave function components. In (**a**), the *x*–*y* boundary of one of the NWs is outlined as a visual reference.

In Fig. 6, we present the ground state distributions for a $6\times 6$ array of 1-micron long PNWs of cross section $L_{x,y}=20$ nm with the mutual separation $L_{b}$ modulated from 8 to 3 nm. From Fig. 6 we can observe the gradual NWs coupling along the *x*–*y* transversal plane as $L_{b}$ becomes smaller. Even when the electronic connection between adjacent NWs in Fig. 6a is not evident, actually for an 8 nm-thick AlGaAs barrier there exist a significant tunneling, for such reason the electronic distribution in each wire in Fig. 6a is affected for the entire NWs array (for a large enough $L_{b}$, each NW is independent from each other and the same electronic *x*–*y* distribution is expected in any NW in the array). Analogous to the 2D case (see Fig. 5b), the electronic distribution is denser at the central part of the array and it decreases for the more external NWs.

**Figure 7:**
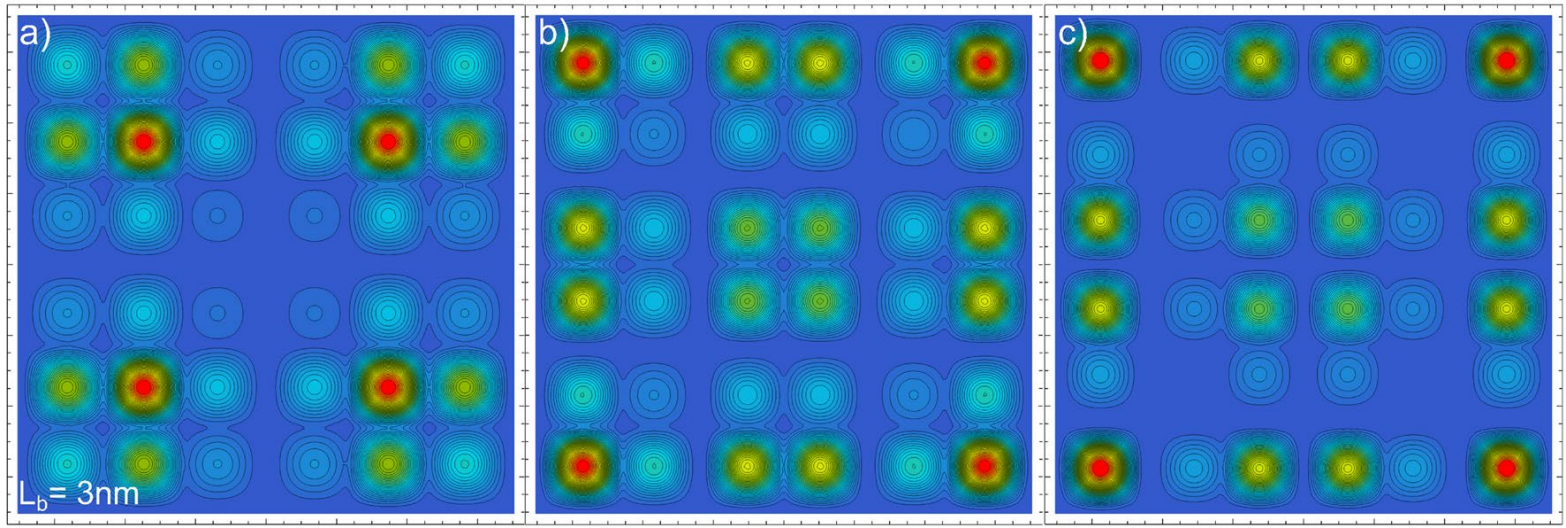
*x*–*y* electron configuration for the (**a**) first, (**b**) second, and (**c**) third excited states in a $6 \times 6$ PNW array with $L_{x,y}=$ 20 nm and $L_b=$ 3 nm. The distributions are obtained by considering only the *x* and *y* wave function components.

Due to the strong coupling between close NWs, collective phenomena can be expected for small $L_{b}$ values. One striking effect is the related to the *x*–*y* excited states, which involves the combined contribution of all the NWs. In Fig. 7 we plot the first three *x*–*y* excited states of the $6 \times 6$ array presented in Fig. 6c ($L_{b}$ = 3nm). As observed, singular electronic distributions are shaped in the NW cross sections, triggering drastic changes in its electronic population. This kind of remarkable PNW collective phenomena described by our model could be used, in analogy to the approach presented in Reference^36^, to deal with more complex problems in two and higher dimensions.

We would like to highlight that such 2D and 3D distributions, together with the possibility to switch between them by means of an external gate, could be of great interest in practical issues as well as in the investigation of new physical phenomena. For example, these NW arrays could be a valuable alternative to usual approaches, such as the 3D arrays of quantum dots to build 3D superlattices^40^ or to substitute the use of different materials along NWs to produce a charge distribution control along the NWs axis^41^. Such well separated charge distributions, at the nanometric scale, could also have applications in the design of alternative 3D NW-based logic architectures^42^.

## Theoretical model

We focus on direct wide bandgap semiconductors, so the small interaction between the conduction and valence bands can be neglected. The model can be easily applicable to others wide bandgap compounds by changing the material parameters. As we use a Yukawa approach, a minimal in the electronic concentration must be fulfilled to guarantee that the $\text {e-e}$ average separation is not larger than the screening length^37^. In this contribution, only suitable nanostructure sizes and *n*-doping levels that satisfy such condition are considered.

The time-independent Schrödinger equation for a spinless two-electron wave function, $\Psi _{1,2} (r) \equiv \Psi _{1,2} ((x_1,y_1,z_1),(x_2,y_2,z_2))$, is

1 $$\begin{aligned} \left[ -\frac{\hbar ^2}{2m_{e}^*} \nabla _{1,2} ^2 +V_{\text {eff}} (r)\right] \Psi _{1,2} (r) =E \Psi _{1,2} (r), \end{aligned}$$

where $\hbar \equiv h/2\pi$, *h* the Planck constant, $m_e^*$ the electron effective mass, and $V_{\text {eff}}$ is the effective potential. $V_{\text {eff}}$, which is derived further below, includes the finite confinement potential in the transversal *x*-*y* plane, the infinite barrier potential at the NW *z*-edges, and the many-body $\text {e-e}$ Yukawa-like interaction.

The Yukawa-like potential is given by

2 $$\begin{aligned} V_{Y}(r)=\frac{e^2}{4\pi \epsilon }\frac{e^{-\kappa r}}{r}, \end{aligned}$$

where $r=\sqrt{(x_1-x_2)^2+(y_1-y_2)^2+(z_1-z_2)^2}$ is the $\text {e-e}$ separation; $\epsilon =\epsilon _0\epsilon _r$ is the absolute permittivity, with $\epsilon _0$ the vacuum permittivity and $\epsilon _r$ the relative permittivity of the material ($\epsilon _r =12.9$ for GaAs and $\epsilon _r=12.247$ for Al$_x$Ga$_{1-x}$As for the Al concentration $x=0.23$ considered in the calculations). The screening parameter $\kappa$ is given by $\sqrt{\frac{2e^2\text {n}}{\epsilon K_{B} T}}$, with *n* the electronic density, $K_B$ the Boltzmann constant, and *T* ($=300$ K) the temperature.

For $L_{x}, L_{y} \le 55$ nm, $L_{b} \le 40$ nm, and $L_{z} \approx$ 1 $\upmu$m, $V_{Y}(r)$ can be considered as a small perturbation in the *x* and *y* directions. Then, we can make an approximation in the left side of Eq. (1) by splitting the transversal ($\perp$) and parallel ($\parallel$) contributions as

3 $$\begin{aligned} H_{\perp }= & {} -\frac{\hbar ^2}{2m_{e}^*} \left( \frac{\partial ^2}{\partial x_1^2}+\frac{\partial ^2}{\partial x_2^2}+\frac{\partial ^2}{\partial y_1^2}+\frac{\partial ^2}{\partial y_2^2}\right)+V_{\text {x-y}}(x,y) \end{aligned}$$

4 $$\begin{aligned} H_{\parallel }= & {} -\frac{\hbar ^2}{2m_{e}^*} \left( \frac{\partial ^2}{\partial z_1^2}+\frac{\partial ^2}{\partial z_2^2}\right) +\frac{e^2}{4\pi \epsilon}\frac{e^{-\kappa r}}{r} \end{aligned}$$

Considering that the wave function is separable in its transversal and longitudinal components as $\Psi _{1,2}({r})=\psi _{\perp _1}(x_1,y_1)\psi _{\perp _2}(x_2,y_2)\psi _{\parallel _{1,2}}(z_1,z_2)$. Then, the *y* component of the wave function for each of the two confined electrons, for an $1 \times m$ PNW array, can be directly calculated from Eqs. (1) and (3) as

5 $$\begin{aligned} \psi _y(y)=N_yCos(k_yy) \end{aligned}$$

where $k_y=\sqrt{\frac{2m_{\text {nw}}^*}{\hbar ^2} E_y}$, $m_{\text {nw}}^* = 0.0665m_e$ for GaAs ($m_e$ the electron mass), and $N_y$ is a normalization constant.

Along the *x*-direction we must solve a system of m-coupled quantum wells, composed of the wave functions in the AlGaAs barriers ($\psi _b(x)$) and the GaAs NW ($\psi _{nw}(x)$):

6 $$\begin{aligned} \psi _{\text {b}_\text {i}}(x)= & {} A_{\text {i}} e^{k_{\text {b}_\text {i}}x}+B_{\text {i}} e^{-k_{\text {b}_\text {i}}x} \end{aligned}$$

7 $$\begin{aligned} \psi _{\text {nw}_\text {j}}(x)= & {} C_{\text {j}}cos(k_{\text {nw}_\text {j}}x)+D_{\text {j}}sin(k_{\text {nw}_\text {j}}x) \end{aligned}$$

in which $\text {i}=1,2,3,\ldots (m+1)$, $\text {j}=1,2,3,\ldots m$, $k_{\text {b}}=\sqrt{\frac{2m_{\text {b}}^*}{\hbar ^2}(V_0-E_x)}$, $k_{\text {nw}}=\sqrt{\frac{2m_\text {nw}^*}{\hbar ^2}E_x}$, $V_0 = 187$ meV, and $m_\text {b}^* = 0.0857m_e$ for Al$_{0.23}$Ga$_{0.77}$As. $A_\text {i}$, $B_\text {i}$, $C_\text {j}$ and $D_\text {j}$ are additional normalization constants.

In order to derive the effective potential, we consider the Fourier transform for each of the cross-sectional wave functions for a single confined electron, in an $1 \times m$ PNW array, given by

8 $$\begin{aligned} \psi _{\text {b}_\text {i}}^2(x)= & {} \frac{1}{2\pi }\int dq_x G_{b_\text {i}}(q_x) e^{-iq_xx} \end{aligned}$$

9 $$\begin{aligned} \psi _{\text {nw}_\text {j}}^2(x)= & {} \frac{1}{2\pi }\int dq_x G_{\text {nw}_{\text {j}}}(q_x) e^{-iq_xx} \end{aligned}$$

10 $$\begin{aligned} \psi _{y}^2(y)= & {} \frac{1}{2\pi }\int dq_y G_{y}(q_y) e^{-iq_yy} \end{aligned}$$

where,

11 $$\begin{aligned} G_{\text {b}_\text {i}}(q_x)= & {} \sqrt{2\pi }\left( \frac{A_{\text {i}}}{B_{\text {i}}}\delta (q_x-2ik_{\text {b}})+\frac{B_{\text {i}}}{A_{\text {i}}}\delta (q_x+2ik_{\text {b}})\right) \nonumber \\&+2\sqrt{2\pi }\delta (q_x),\nonumber \\ \end{aligned}$$

12 $$\begin{aligned} G_{\text {nw}_\text {j}}(q_x)&= {} \sqrt{\frac{\pi }{2}}\left[ \frac{C_{\text {j}}^2+D_{\text {j}}^2}{C_{\text {j}}D_{\text {j}}}\delta (q_x)\right]+ \sqrt{\frac{\pi }{2}}\frac{C_{\text {j}}^2-D_j^2}{C_{\text {j}}D_{\text {j}}}\left[ \frac{\delta (q_x-2k_{\text {nw}})}{2}+\frac{\delta (q_x+2k_{\text {nw}})}{2}\right] \nonumber \\&\quad + \sqrt{\frac{\pi }{2}}i\left[ \delta (q_x-2k_{\text {nw}})-\delta (q_x+2k_{\text {nw}}\right] , \end{aligned}$$

13 $$\begin{aligned}G_{y}(q_y)= \sqrt{\frac{\pi }{2}}N_y\left[ \delta (q_y)+\frac{\delta (q_y-2k_{y})}{2}+\frac{\delta (q_y+2k_{y})}{2}\right] .\nonumber \\ \end{aligned}$$

For the longitudinal component, the corresponding Fourier transform is

14 $$\begin{aligned} \psi ^2_z(z)=\frac{1}{(2\pi )^2}\int dq_z G_{z}(q_z) e^{-iq_zz} \end{aligned}$$

Then, by replacing Eqs. (8–10) and (14) into the $\text {e-e}$ interaction energy, in its integral representation:

15 $$\begin{aligned} W= \int d^3r_1d^3r_2 \left( \frac{e^2}{4\pi \epsilon }\frac{e^{-\kappa r}}{r} \right) | \Psi _{1,2} (\mathbf {r})|^2, \end{aligned}$$

where

16 $$\begin{aligned} \frac{e^{-\kappa r}}{r}=\frac{1}{2\pi ^2}\int d^3q \frac{e^{-i(q_xx+q_yy+q_zz)}}{\kappa ^2+q_x^2+q_y^2+q_z^2}, \end{aligned}$$

We can now consider the contribution of both electrons and, defining $G(q_x,q_y)=G_{\text {nw}_\text {j}/\text {b}_\text {i}}(q_x)G_y(q_y)$, we obtain

17 $$\begin{aligned} W= \frac{e^2}{4\pi \epsilon _{\text {nw}/\text {b}}2^7\pi ^8}\int d^3r\int d^3q \frac{G^2(q_x,q_y)G_{z}(q_z) }{\kappa ^2+q_x^2+q_y^2+q_z^2}\nonumber \\ \times e^{-i(q_xx+q_yy+q_zz)} \end{aligned}$$

where $d^3q\equiv dq_xdq_ydq_z$ and $d^3r \equiv dxdydz$; $\epsilon _{\text {nw}/\text {b}}$ corresponds to the permittivity that is replaced for each corresponding region (NW or the barrier) and $G_{\text {nw}/\text {b}}$ is defined analogous to the permittivity. Integrating over *r*,

18 $$\begin{aligned} W=\frac{e^2}{4\pi \epsilon _{\text {nw}/\text {b}} 2\pi ^2}\int d^3q \frac{G^2(q_x,q_y)G_{z}(q_z) }{\kappa ^2+q_x^2+q_y^2+q_z^2} \end{aligned}$$

Now, as

19 $$\begin{aligned} G_z(q_z)=\int dz\psi ^2_z(z)e^{\pm i(q_zz)}, \end{aligned}$$

Equation (18) can be expressed as

20 $$\begin{aligned} W=\frac{e^2}{4\pi \epsilon _{\text {nw}/\text {b}} 2\pi ^2}\int dz d^3q\frac{G^2(q_x,q_y)\psi _z^2(z)e^{iq_zz} }{\kappa ^2+q_x^2+q_y^2+q_z^2} \end{aligned}$$

Then, by using the equality

21 $$\begin{aligned} \int \limits _{-\infty } ^{\infty } dq_z \frac{e^{iq_zz}}{q_x^2+q_y^2+q_z^2+\kappa ^{2}}=\frac{\pi e^{-|z|\sqrt{q_x^2+q_y^2+\kappa ^{2}}}}{\sqrt{q_x^2+q_y^2+\kappa ^{2}}}, \end{aligned}$$

Equation (20) can be rewritten as

22 $$\begin{aligned} W=\frac{1}{2\pi } \int \limits _{-\infty } ^{\infty } dz\psi _z^2(z)V_{\text {eff}}(z), \end{aligned}$$

where $V_{\text {eff}}$ is defined by

23 $$\begin{aligned} V_{\text {eff}}(z)= \frac{e^2}{4\pi \epsilon _{\text {nw}/\text {b}}}\int \limits _{l} ^{\infty } dq_xdq_yG^2(q_x,q_y)\frac{e^{-|z|\sqrt{q_x^2+q_y^2+\kappa ^{2}}}}{\sqrt{q_x^2+q_y^2+\kappa ^{2}}} \end{aligned}$$

By replacing Eqs. (11–13) in (23), we can obtain the final expression for $V_{\text {eff}}$, which must be evaluated in each part of the GaAs/AlGaAs NWs along the *z*-direction. Then, for the extreme left and right AlGaAs barriers,

24 $$\begin{aligned} V_{\text {eff}_\text {b}}= \frac{\left[ N_y \pi e \right] ^2}{8 \pi \epsilon _\text {b}} \left[ A_\text {i}^2 + B_\text {i}^2 \right] \left[ \frac{e^{-|z|\sqrt{4k_{\text {b}}^2+4k_{y}^2+\kappa ^2}}}{\sqrt{4k_{\text {b}}^2+4k_{y}^2+\kappa ^2}} \right] , \end{aligned}$$

at the internal barriers,

25 $$\begin{aligned} V_{\text {eff}_\text {b}}= \frac{\left[ N_y\pi e\right] ^2}{8\pi \epsilon _\text {b}}\left[ \frac{A_\text {i}^4+B_\text {i}^4}{A_\text {i}^2B_\text {i}^2}\right] \left[ \frac{e^{-|z|\sqrt{4k_{\text {b}}^2+4k_{y}^2+\kappa ^2}}}{\sqrt{4k_{\text {b}}^2+4k_{y}^2+\kappa ^2}} \right] , \end{aligned}$$

and, in the GaAs NWs,

26 $$\begin{aligned} V_{\text {eff}_{\text {nw}}}= \frac{\left[ N_y\pi e\right] ^2}{64\pi \epsilon _{\text {nw}}} \frac{\left[ C_\text {j}^2+ D_\text {j}^2\right] ^2}{C_\text {j}^2 D_\text {j}^2} \left[ \frac{e^{-|z|\sqrt{4k_{\text {nw}}^2+4k_{y}^2+\kappa ^2}}}{\sqrt{4k_{\text {nw}}^2+4k_{y}^2+\kappa ^2}} \right] , \end{aligned}$$

To solve the Schrödinger equation by using Eqs. (24), (25), and (26), we use the finite difference method.

On the other hand, for a $m_1 \times m_2$ array of PNWs we can follow a similar procedure but, instead of Eq. (5) for the *y* component, we must consider Equations of the type (6) and (7). Following the procedure described before, the final effective potentials for these arrays are, at the external barriers,

27 $$\begin{aligned} V_{\text {eff}_\text {b}}= \frac{\left[ 2\pi e \right] ^2}{4 \pi \epsilon _\text {b}} \left[ A_\text {i}^2 + B_\text {i}^2 \right] \times \left[ O_\text {i}^2 + P_\text {i}^2 \right] \left[ \frac{e^{-|z|\sqrt{4k_{\text {b}}^2+4k_{y}^2+\kappa ^2}}}{\sqrt{4k_{\text {b}}^2+4k_{y}^2+\kappa ^2}} \right] , \end{aligned}$$

at the internal barriers,

28 $$\begin{aligned} V_{\text {eff}_\text {b}}= \frac{\left[ 2\pi e\right] ^2}{8\pi \epsilon _\text {b}}\left[ \frac{A_\text {i}^4+B_\text {i}^4}{A_\text {i}^2B_\text {i}^2}\right] \times \left[ \frac{O_\text {i}^4+P_\text {i}^4}{O_\text {i}^2P_\text {i}^2}\right] \left[ \frac{e^{-|z|\sqrt{4k_{\text {b}}^2+4k_{y}^2+\kappa ^2}}}{\sqrt{4k_{\text {b}}^2+4k_{y}^2+\kappa ^2}} \right] , \end{aligned}$$

and, in the GaAs NWs,

29 $$\begin{aligned} V_{\text {eff}_{\text {nw}}}= \frac{\left[ \pi e\right] ^2}{64\pi \epsilon _{\text {nw}}} \frac{\left[ C_\text {j}^2+ D_\text {j}^2\right] ^2}{C_\text {j}^2 D_\text {j}^2} \times \frac{\left[ Q_\text {j}^2+ R_\text {j}^2\right] ^2}{Q_\text {j}^2 R_\text {j}^2} \left[ \frac{e^{-|z|\sqrt{4k_{\text {nw}}^2+4k_{y}^2+\kappa ^2}}}{\sqrt{4k_{\text {nw}}^2+4k_{y}^2+\kappa ^2}} \right] . \end{aligned}$$

where $O_\text {i}$, $P_\text {i}$, $Q_\text {j}$ and $R_\text {j}$ are the additional normalization constants related to the *y* component.

## Conclusion

We present theoretical calculations of two-electron states under the influence of a variable electron screening in semiconductor parallel NWs, considering the effect of the system size, the *n*-doping level and the NWs separation. When a low-density Wigner crystal regime is considered, localized effects in the tunneling between adjacent quantum wires are observed. By modifying NWs parameters such as the cross-section, the *n* concentration and the NWs separation, the charge distribution pattern in 2D and 3D PNW arrays can form interconnected distributions between the adjacent NWs. Such nanoscale localized charge distribution could be valuable in the design of new architectures for photonics and electronics applications.
